# Support to woman by a companion of her choice during childbirth: a randomized controlled trial

**DOI:** 10.1186/1742-4755-4-5

**Published:** 2007-07-06

**Authors:** Odalea M Bruggemann, Mary A Parpinelli, Maria JD Osis, Jose G Cecatti, Antonio S Carvalhinho Neto

**Affiliations:** 1 Department of Nursing, Federal University of Santa Catarina, Florianopolis, SC, Brazil; 2 Department of Obstetrics and Gynecology, School of Medical Sciences, University of Campinas (UNICAMP), São Paulo, Brazil; 3 Center for Research in Reproductive Health of Campinas (CEMICAMP), São Paulo, Brazil; 4 State Hospital of Sumare, University of Campinas, São Paulo, Brazil

## Abstract

**Background:**

To evaluate the effectiveness and safety of the support given to women by a companion of their choice during labor and delivery.

**Methods:**

A total of 212 primiparous women were enrolled in a randomized controlled clinical trial carried out between February 2004 and March 2005. One hundred and five women were allocated to the group in which support was permitted and 107 to the group in which there was no support. Variables regarding patient satisfaction and events related to obstetrical care, neonatal results and breastfeeding were evaluated. Student's t-test or Wilcoxon's test, chi-square or Fisher's exact test, risk ratios, and their respective 95% confidence intervals were used in the statistical analysis.

**Results:**

Overall, the women in the support group were more satisfied with labor (median 88.0 versus 76.0, p < 0.0001) and delivery (median 91.4 versus 77.1, p < 0.0001). During labor, patient satisfaction was associated with the presence of a companion (RR 8.06; 95%CI: 4.84 – 13.43), with care received (RR 1.11; 95%CI: 1.01 – 1.22) and with medical guidance (RR 1.14 95%CI: 1.01 – 1.28). During delivery, satisfaction was associated with having a companion (RR 5.57, 95%CI: 3.70 – 8.38), with care received (RR 1.11 95%CI: 1.01 – 1.22) and with vaginal delivery (RR 1.33 95%CI:1.02 – 1.74). The only factor that was significantly lower in the support group was the occurrence of meconium-stained amniotic fluid (RR 0.51; 95%CI: 0.28 – 0.94). There was no statistically significant difference between the two groups with respect to any of the other variables.

**Conclusion:**

The presence of a companion of the woman's choice had a positive influence on her satisfaction with the birth process and did not interfere with other events and interventions, with neonatal outcome or breastfeeding.

## Background

The rates of maternal and neonatal mortality and morbidity decreased as a consequence of the adoption of modern obstetric practices, especially during labor and delivery. However, obstetrical interventions continued to increase, particularly the rate of Caesarean sections. Active management is based on the assumption that the preventive management of events that may potentially result in adverse effects in the mother or the fetus reduces the morbidity rates of both [1].

Support provided during labor and delivery by professional healthcare workers, non-medical female attendants and trained women (*doulas*) assigned to this task has been evaluated in controlled studies [2]. Data suggest that the effects of support are associated with a reduction in the dissatisfaction or negative perception of women towards giving birth, in the use of analgesia/anesthesia, and in the frequency of instrumental vaginal delivery (forceps and vacuum extraction) and Caesarean section [3].

Based on scientific evidence, the World Health Organization recommends that the parturient should be accompanied by people she trusts and with whom she feels at ease, possibly her partner, a friend, a *doula*, a nurse or midwife [4]. However, the effects of the support provided by the presence of the woman's chosen companion on her satisfaction, on the events of labor and delivery and on perinatal results have not yet been fully evaluated in controlled studies [5,6]. The usefulness of support and the type of support provided by family members, a partner or by friends of the woman have only been evaluated in observational studies [2,3].

It is important to recognize and understand the influence of such support not only because of its effect on obstetrical and perinatal events but also on the patient's attitude towards the birth experience itself. Although, since 2005, following some initial isolated state initiatives, it is guaranteed by national law to all Brazilian women to have a companion of her choice present during labor, it is not respected by many services and providers [5]. Due to the paucity of evidence-based data available on the effects of the presence of a companion of the woman's choice during the birth process, especially in developing countries, this study was developed to evaluate the influence of this support provider on the satisfaction of the parturient with labor and delivery and on perinatal and breastfeeding outcomes in the twelve hours following delivery.

## Methods

A randomized controlled trial was carried out between February, 2004 and March, 2005 at the Sumare maternity hospital linked with the University of Campinas, São Paulo, Brazil. Sample size was based on a previous study in which the support given by nurses during delivery was evaluated [6]. Considering a difference of 15.1% between the groups regarding patient satisfaction, a significance level of 5% and a power of 80%, minimum sample size was calculated at 96 patients in each group. Considering a possible loss of information or discontinuation of up to 10%, total sample size was calculated at 212 women.

Inclusion criteria were: primiparous pregnant women with a single, term live cephalic fetus; in active labor – cervical dilation ≥3 cm and ≤6 cm; intact membranes or amniorrhexis of ≤2 hours; uterine height < 40 cm; no evidence of cephalic-pelvic disproportion or fetal distress. Exclusion criteria were: unavailability of a companion; fetal malformation; maternal disease and/or indication for elective Caesarean section.

The study was approved by the Institutional Review Board and by the director of the hospital. At the time of the study for women to have a companion during labor was not a policy at that institution, as it is still not for the majority of institutions in Brazil. Therefore, to participate in such study would theoretically represent a potential benefit for the women. The eligible women and their chosen companions were supplied with information on the objectives and design of the study, and agreed to participate by signing an informed consent form.

Randomization was carried out using a computer-generated sequence of 212 random numbers. The individual assignment numbers were all placed in an opaque container to assure the concealment. The eligible women who had agreed to participate in the study selected one of the numbers once, and were therefore allocated either to the intervention group (with support) or to the control group (no support) according to the list. Support was defined as presence of a chosen companion during labor and delivery.

In both groups, care during labor and delivery was provided according to the routine protocol of the institution, including active management of labor, a relatively common procedure in Brazilian maternities: early amniotomy, use of oxytocin, intermittent electronic fetal monitoring, and systematic analgesia. At this institution, a companion during labor and delivery had not previously been permitted. This was the only difference between the two groups.

The companions received standardized verbal and written instructions provided by the principal investigator, containing information on: the activities involved in providing support to the woman (stay beside her, provide support, be affectionate, keep her calm, massage her, stimulate and encourage her), expected behavior when confronted with signs of tiredness, anxiety, concern, crying, screaming and/or the woman's feelings of inability to cope; compliance with regulations (use of standardized clothing, no eating, no smoking, no touching the equipment or material, contact the nursing staff if need to leave); and the possibility of requesting information from staff. The need to preserve the privacy of the other women was also emphasized. There were no specific instructions for the health professionals.

The outcomes included satisfaction, assessed by asking the woman about how she felt during labor and delivery (evolution of labor, having a companion or not, instructions received from doctors and nursing staff, healthcare provided and type of delivery). These questions were answered by choosing one of a sequence of five symbols with facial expressions corresponding to "very dissatisfied", "dissatisfied", "satisfied", "well satisfied" and "very satisfied". Satisfaction assessment was carried out between 12–24 hours post delivery at rooming-in care unit. For the purpose of analysis, satisfaction was considered to have been achieved whenever the answers of "well satisfied" or "very satisfied" were given [7,8]. We collected data on the following outcomes: duration of first stage of labor; amniotomy in relation to the time of hospital admission and cervical dilation; color of amniotic fluid; use of oxytocin in relation to cervical dilation; time of analgesia in relation to cervical dilation and time of admission to hospital; presence of functional dystocia and changes in fetal wellbeing; length of the second stage; time between hospital admission and delivery; time from analgesia until delivery; type of delivery (vaginal/Caesarean). Neonatal outcomes were: Apgar score at 1 and 5 minutes, birthweight, admission to the neonatal intensive care unit (NICU), and immediate mother-infant contact following delivery. Variables regarding breastfeeding were: the ability of the infant to take the breast and suckling in the delivery room and in the 12 hours following delivery, cracked nipples and the number of breast-feeds in the first 12 hours.

We used SAS software program, version 8.2 for statistical analysis. An intention-to treat-analysis was performed. Mean and medians were calculated for continuous variables, while Student's t and Wilcoxon tests were used to assess differences between groups. For categorical variables, chi-square or Fisher's exact tests were used. Risk ratios and their respective 95% confidence intervals were calculated for the main outcomes. Significance was established as p < 0.05.

## Results

**Figure 1:**
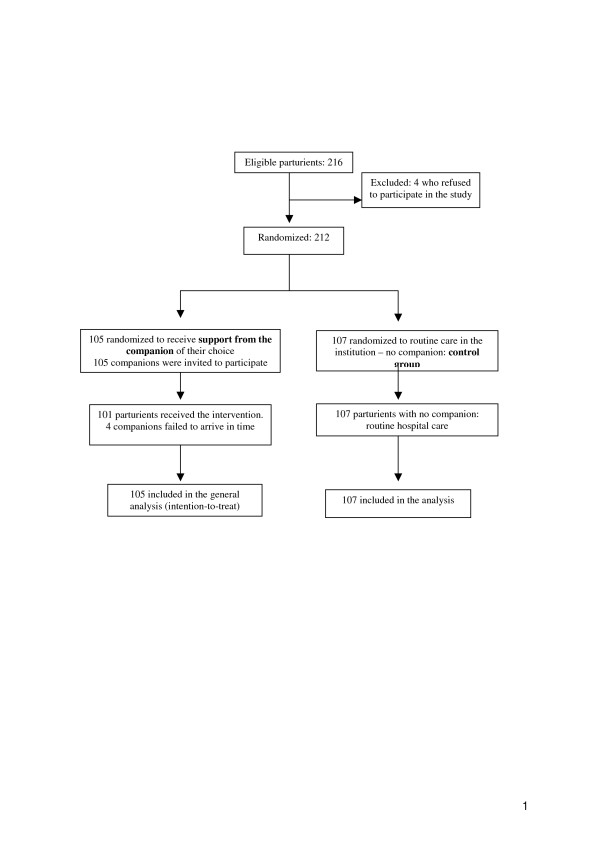
Flowchart of participants through trial.

A total of 212 parturients participated in the study, 105 in the intervention group and 107 in the control group (Figure 1). From a total of 105 companions, most common was the woman's partner/father of the child (47.6%), followed by the woman's mother (29.5%) or another female relative (aunt, mother-in-law, sister, cousin, sister-in-law, grandmother) or friend (22.8%). A total of 49.5% of companions were already present when the parturient was admitted to hospital, while 50.5% were located and invited to participate by telephone. The mean age of companions in this study was 33.5 years (range 18–62 years). Most (68.3%) had primary education and 71.3% had paid employment. Their support was provided continuously and they left the woman's side only sporadically.

Table 1 shows that there were no significant differences between the groups in sociodemographic and obstetrical characteristics of women at the time of hospital admission. Regarding satisfaction with the birth experience, having a companion during labor and delivery were strongly associated with higher satisfaction in the intervention group. The women of this group were also more satisfied with the care they received during labor, with the medical guidance given during labor, with care received during delivery, and with vaginal delivery, than women in the control group (Table 2).

**Table 1:** Baseline sociodemographic and obstetrical characteristics of the women, according to group

**Characteristic**	**Support** **(n = 105)**	**Control** **(n = 107)**
Age [mean in years (range)]	20.6 (13–42)	20.1 (14–36)
In a stable union (n)	80 (76.2%)	92 (85.9%)
Secondary education (n)	103 (98.1%)	106 (99.1%)
Religious (n)	96 (91.4%)	100 (93.4%)
Non-white skin color (n)	79 (75.2%)	75 (70.1%)
Housewife (n)	63 (60.0%)	67 (62.6%)
Start of prenatal care (GA < 28 weeks) (n)	101 (96.2%)	103 (96.3%)
Number of prenatal visits ≥ 6 (n)	84 (85.0%)	84 (78.5%)
Accompanied during prenatal care (n)	44 (41.9%)	54 (50.5%)
Participated in classes for pregnant women (n)	16 (15.2%)	16 (14.9%)
Gestational age at delivery [mean in weeks (range)]	39.2 (37–42)	39.0 (37–42)
Cervical dilation at admission [mean in cm (range)]	3.9 (3–6)	3.9 (3–6)
Cervical effacement ≥ 80% (n)	75 (71.4%)	68 (63.6%)
Intact amniotic membrane (n)	82 (78.1%)	83 (77.6%)

There were no statistically significant differences between the groups.

**Table 2:** Risk ratios and 95% confidence intervals for satisfaction ("well satisfied" or "very satisfied") during labor and delivery, according to group

**Variable**	**Support** **(n = 105)**	**Control** **(n = 107)**	**RR** **(95%CI)**	**p value**
**Labor**				
Evolution of labor	56	49	1.16 (0.89–1.53)	0.272
Having a companion	96	13	10.08 (5.38–18.89)	<0.0001*
Care received	98	90	1.11 (1.01–1.22)	0.034
Medical guidance	94	84	1.14 (1.01–1.28)	0.028
Guidance from nursing staff	94	89	1.08 (0.97–1.20)	0.178
**Delivery**				
Evolution	73	60	1.24 (1.00–1.53)	0.042
Having a companion	95	19	8.17 (4.51–14.78)	<0.0001*
Care received	98	90	1.11 (1.01–1.22)	0.034
Medical guidance	91	86	1.08 (0.96–1.22)	0.217
Guidance from nursing staff	93	92	1.13 (0.72–1.77)	0.571
Type of delivery				
Vaginal	58	44	1.33 (1.02–1.74)	0.033
Caesarean	2	6	0.40 (0.10–1.40)	0.193*

Chi-squared test, *Fisher's Exact Test

The occurrence of meconium-stained amniotic fluid was the only obstetrical outcome related to labor or delivery that was statistically significantly lower in the intervention compared to the control group (RR 0.51; 95%CI: 0.28 – 0.94), (Table 3). Regarding the newborn and breastfeeding outcome, there were no statistically significant differences between the intervention and control groups (Table 4).

**Table 3:** Effects of intervention on the events of labor and delivery, according to group

**Event**	**Support** **(n = 105)**	**Control** **(N = 107)**	**RR** **(95% CI)**	**p value**
**Cervical dilation **[median (range)]				
Amniotomy	5 (3–8)	5 (3–10)	-	0.958^†^
Oxytocin	4 (3–9)	4 (3–9)	-	0.653^†^
Analgesia	5 (3–10)	5 (3–10)	-	0.253^†^
**Functional Dystocia **				
Absent	99	97	-	0.655
Tachysystole	2	3	0.66 (0.11–3.87)	
Hypo/oligo-systole	4	7	0.58 (0.17–1.91)	
**Color of amniotic fluid**				
Clear	91	80	-	0.020
Meconium-stained	13	26	0.51 (0.28–0.94)	
**Fetal heart rate**				
Unaltered	81	76	-	
Altered	24	31	1.18 (0.84–1.66)	0.309
**Type of delivery**				
Vaginal	94	95	-	
Caesarean	11	12	0.93 (0.43–2.02)	0.862
**Time **[median (range)]				
First stage of labor^§ ^(h)	3.4 (1.2–15.5)	3.8 (1.4–11.8)	-	0.123^†^
Admission – amniotomy (h)	1.1 (0–6.9)	1.2 (0–9.0)	-	0.639^†^
Admission – analgesia (h)	1.7 (0.1–7.8)	1.8 (0.3–9.3)	-	0.283^†^
Second stage of labor^§ ^(min)	18 (4.8 – 75)	16.2 (1.2–48)	-	0.368^†^
Analgesia – birth (h)	2.3 (0.1–14.6)	2.3 (0.1–8.6)	-	0.605^†^
Hospital admission – birth (h)	3.8 (1–16)	4.3 (1.3–12.2)	-	0.284^†^

^†^Wilcoxon test, ^§^Caesarean sections excluded, Chi-square test

**Table 4:** Effects on the newborn infant and breastfeeding outcomes, according to group

**Outcomes**	**Support (N = 105)**	**Control (N = 107)**	**RR (95%CI)**	**p value**
Apgar score at 1 minute < 7	20	21	0.97 (0.56–1.68)	0.915
Apgar score at 5 minutes < 7	3	2	1.53 (0.26–8.96)	0.681*
Birthweight (g) (mean ± SD 95%)	3.197 (2.360–4.245)	3.246 (2.410–4.145)	-	0.370^¶^
Admission to NICU	5	6	0.91 (0.47–1.77)	0.781
Immediate contact mother/newborn	52	41	1.29 (0.95–1.76)	0.100
Time of contact mother/newborn (min) (mean ± 95% SD)	25.1 (10–55)	22.7 (10–40)	-	0.360^†^
Takes breast/suckles in delivery room	12	7	1.75 (0.72–4.26)	0.213
Takes breast/suckles (12 h following birth)	99	100	1.08 (0.59–1.97)	0.801
Breast fissure	7	6	1.19 (0.41–3.42)	0.747
Number of breast-feeds 12 hours following birth (mean)	4.3 (0–12)	4.4 (0–10)	-	0.589^†^

Chi-squared test, * Fisher's Exact Test, ^¶^Student's t-test, ^† ^Wilcoxon's test

## Discussion

These results show that the support provided by a companion of the woman's choice during labor and delivery had a positive effect on her satisfaction with the birth experience. Although the opinion of the health professionals were not assessed systematically, it seems that this intervention was well-accepted by them. No previous training was offered to the health workers, and the companions underwent no prior preparation. Therefore, the assistance the women in both groups received during labor and delivery was the standard care routinely provided in that hospital, and there were no changes in management. It is important to emphasize that this is not a study about *doula*s and if on one hand there is a general belief that a labor companion has always positive effects, there are,, on the other hand still a lot of health facilities where companions are not allowed, especially in developing settings. It was and still it is expected that the results of this study could help providers to acknowledge and respect women's rights during birth.

Satisfaction may have been influenced by assessment in the first 12–24 hours postpartum, in which feelings of dependency and benevolence and a halo effect are common. This effect describes a lack of criticism due to social ability and/or fear of reprisals, or because of a sensation of relief at having gone through a safe experience and having a healthy baby [9,10]. However, this effect would probably be the same for both groups and could not explain the difference between them.

Experience during birth has been evaluated in controlled studies in which the type of care provider (*doula*, nurse or lay-person) varied. In most cases, anxiety, self-esteem, feelings of failure and difficulty, as well as levels of personal control and pain were assessed [11,12]. In the present study, a chosen companion was the most important factor affecting the satisfaction of the parturient with labor and delivery, similar to what was found by Bertsch et al. [13]. In other controlled studies the presence of a partner or other family member [12,14,15] was not permitted or it was already a common practice in the institution [6,16,17] and was therefore not evaluated. These findings differ from those of Langer et al. [15], who reported that support had no influence on women's satisfaction in a study in which the presence of family members was not allowed and the majority of *doulas *were retired nurses.

In the intervention group, women's greater satisfaction with the guidance received from the doctors during labor has also been identified in another study with a different population, evaluated when the woman was accompanied by a person of her choosing [18]. When *doulas *or professional healthcare workers are the support providers, instructions are generally supplied by these individuals [9,15-17]. Support also increased satisfaction with the care received during labor and delivery, and this finding is in agreement with data already reported [6] when the women received support from nurses.

Support also contributed towards satisfaction with vaginal delivery. Similar results were reported in other studies where women in the control group considered the experience of giving birth worse than they had imagined, compared to those in the intervention group [11,19]. Therefore, it would appear that the presence of a person specifically designated to provide support positively influences the woman's perception of the birth experience itself, as seen in some meta-analysis and systematic reviews [5,20]. This higher level of satisfaction may have been influenced by the woman's expectations and the way in which she perceived her care and by having a companion in a setting in which normally this would not be permitted.

Similar conclusions may also be drawn with respect to pain, which is considered a great generator of dissatisfaction. In our study, however, all the women were submitted to analgesia during labor. It would appear that the influence of pain and pain relief on satisfaction is not as obvious, direct or beneficial as the influence of the attitudes and behavior of professional health workers [9]. Further studies are required to investigate the influence of pain on satisfaction [3,9].

The finding of a lower occurrence of meconium-stained amniotic fluid may be due to a possible reduction in the anxiety of women who received support, although this was not measured. It is known that an elevated level of maternal epinephrine resulting from stress affects blood flow to the fetus through an α-adrenergic constrictive effect on uterine vascularization, causing transitory hypoxia [21]. On the other hand, emotional support and the measures of comfort and information provided to the woman may reduce her anxiety and fear [4].

The lack of effect of support on any of the other events may have been due to the nature of the study protocol, in which active management of labor was adopted, as it is relatively common in a great proportion of Brazilian maternities, although not confirmed as a real effective intervention. This possible bias may have minimized the positive effects of support on some of the outcomes. This makes the finding of less lower occurrence of meconium-stained fluid even more important, possibly reflecting the positive stress-prevention aspect of support in labor in its potential impact over the newborn. This data is in agreement with results from a multicentric study carried out by Hodnett et al. [6] in which support was provided by nurses. The benefits of support may be surpassed by the rates of intervention carried out in the environment in which delivery occurs; routine analgesia being the factor that most reduces the effect of support on obstetrical interventions [4].

The results regarding the duration of the first stage of labor are contradictory to data reported from studies in which support was provided by lay-women [12], *doulas *[15] and midwives [22], where it was reduced. However, it must be considered that in our study first stage of labor was short in both groups. With respect to Caesarean section, it is noteworthy that rates were low in both groups, and there was no effect of labor support on these rates. This finding is in conflict with reports from other studies [12,14,23] in which the rate of Caesarean section was lower in the group receiving support.

In general, support had no effect on the management of labor in the institution. Interventions such as the use of oxytocin, amniotomy and analgesia, when evaluated in relation to cervical dilation, were carried out early in both groups, and the time between hospital admission, analgesia and amniotomy was less than two hours. Intervention had also no influence on neonatal outcomes and these data are in agreement with other trials [6,11,15,17]. In this study, results regarding breastfeeding were similar in the two groups; however, breastfeeding was only analyzed in the first twelve hours following delivery, while ideally it should be evaluated the first months following delivery [11,15].

## Conclusion

One important finding of this study is that a lay-companion in places where its presence had not previously been permitted has no effect on the routine of care. The fact that the women with support reported higher levels of satisfaction with the medical information/guidance they received indicates that perhaps there was a change in attitude. Perhaps because there was someone else in the room, medical staff were more forthcoming and user-friendly than when no support person was present. These findings of higher patient satisfaction may also encourage and sensitize healthcare providers to adopt this practice in health institutions where such a support companion is not permitted, or even where *doulas*, lay-persons or professional healthcare providers are designated to this role.

In this context, this study may provide a basis for the planning and execution of actions aimed at implementing this practice. Moreover, it may contribute towards increasing the value of the presence of a companion of the woman's choice. Additionally this type of support incurs no extra onus to the institution or to the woman. Therefore, socioeconomic status is not a factor that would limit or impede the implementation of this action. Both the women and the healthcare providers may benefit from this practice, since support improved maternal satisfaction with the birth process, and consequently benefits all those involved in this process. This hopefully could be an advertisement to all places where women still deliver alone.

## Abbreviations

NICU: neonatal intensive care unit

RR: Risk Ratio

95%CI: 95% Confidence Interval

## Competing interests

The author(s) declare that they have no competing interests.

## Authors' contributions

OMB and MAP participated in all the steps of the study, including the project planning, data collection, data analysis and writing the manuscript. MJDO and JGC participated in the project planning and review of the manuscript. ASCN participated in data collection and in writing the final report. All authors provided suggestions for the manuscript, read it carefully, agreed on its content and approved the final version.
